# Association of social isolation with health status among community-dwelling Chinese older adults living with homecare services: a cross-sectional survey in Hong Kong

**DOI:** 10.3389/fpubh.2023.1099734

**Published:** 2023-06-02

**Authors:** Eliza Lai-Yi Wong, Hong Qiu, Annie Wai-Ling Cheung, Hera Hiu-Wah Leung, Frank Youhua Chen, Eng-Kiong Yeoh

**Affiliations:** ^1^Center for Health Systems and Policy Research, JC School of Public Health and Primary Care, Faculty of Medicine, The Chinese University of Hong Kong, Shatin, Hong Kong SAR, China; ^2^JC School of Public Health and Primary Care, Faculty of Medicine, The Chinese University of Hong Kong, Shatin, Hong Kong SAR, China; ^3^Department of Management Sciences, City University of Hong Kong, Kowloon, Hong Kong SAR, China

**Keywords:** multi-criteria decision analysis, social isolation, health status, old adults, cross-sectional survey

## Abstract

**Background:**

Defined as having few social relationships or infrequent social contact with family, friends, and the community, social isolation is a public health crisis. We aimed to evaluate the prevalence of social isolation and explore the association between social isolation and health status among community-dwelling Chinese Older Adults living with homecare services.

**Methods:**

This is a cross-sectional survey with a structured questionnaire conducted among older adults aged ≥60 in the Central Kowloon District of Hong Kong during 2017–2018. Social isolation was assessed by the Lubben Social Network Scale-6 and a score less than 12 was defined as socially isolated. Six aspects of health status including fall risk, cognitive function, depression, activities of daily living (ADL), instrumental activities of daily living (IADL), and functional mobility were measured by standardized instruments. Multi-criteria decision analysis (MCDA) was applied to estimate an index to represent the overall health status of the respondents. Multivariate logistic/linear regression models were applied to examine the associations between social isolation and health status after adjusting the sociodemographic characteristics.

**Results:**

Among the 1,616 participants included in this analysis, the mean age was 80.9 years, 66.3% were female and 41.4% were identified as socially isolated. Compared with the non-isolated group, the socially isolated group had higher proportions of males, divorced or unmarried, ever smoking and drinking, living alone, and living in public housing without religion. After adjusting for confounders, the odds ratios (OR) comparing the socially isolated vs. non-isolated groups were 2.52 (95%CI: 1.79, 3.56) for high fall risk, 1.51 (1.17, 1.94) for cognitive impairment, and 1.78 (1.31, 2.43) for depression. The socially isolated group increased the odds of abnormal ADL, IADL, and functional mobility by 105–150%, and decreased the overall health score by 5.30 (3.42, 7.18).

**Conclusion:**

We demonstrated the association of social isolation with poorer physical function and mental health and overall health status among the community-dwelling Chinese older adults living with homecare services. These findings provided new knowledge about the association of social isolation with both physical and mental function for daily living even for those receiving an integrated homecare service in the community. It implies that an unmet healthcare need existed when comparing the service scope of the current homecare services in the community. It also highlighted the need for targeted prevention and intervention initiatives among community-dwelling old adults to alleviate social isolation for better health and good functioning in the community.

## Introduction

Defined as having few social relationships or infrequent social contact with family, friends, and the community—social isolation is a public health crisis. Since the COVID-19 pandemic began worldwide in 2020, more people experienced social isolation (1–4). Social isolation has been identified as an important social determinant that is associated with adverse health outcomes and a growing public health concern and priority in the post-COVID world (5). Social isolation is known to have negative effects on physical and mental health, especially in older adult populations. For example, one United Kingdom study with a group of middle-aged and older adults (50 years or more) found a relationship between social isolation and poor health status/health-related quality of life (1). Another longitudinal cohort study in older adults in the United Kingdom found social isolation at baseline was associated with higher odds of depressive disorder and poor physical capability in follow-up years (2). The greater social isolation in older men and women related to reduced everyday objective physical activity and greater sedentary time was also reported in the English Longitudinal Study of Aging (6).

There comes a viewpoint that the number of lives lost from social isolation, unemployment, and psychological consequences of different policy decisions would outweigh the number of lives lost from the severe acute respiratory syndrome coronavirus2 (SARS-CoV-2) infection (7). Studies confirm that social isolation is associated with physical health (2, 8), physical activity (9–11), mental health (12–16), and quality of life (1, 2, 6, 17). Nowadays, different kinds of social care supports are provided in the community to improve the daily living and healthcare of older adults, homecare services would be one of the popular community care in Hong Kong (18). Older adults who lived alone or lacked family support may receive homecare services from non-governmental organizations (NGOs) according to their needs, including meal delivery services, escort services, personal care, etc. However, studies about the prevalence of social isolation and its association with physical function at the family and societal level among the social care receivers in the elder population with deteriorated physical health are scanty.

We conducted the current epidemiological cross-sectional study to examine the prevalence of social isolation and its association with physical function, mental disorders, and overall health status among older adult’s recipients of homecare services in Hong Kong, as no study had targeted this vulnerable population who were older, in worse health, and may be more socially isolated. Physical functions were assessed including fall risk, activities of daily living (ADL), instrumental activities of daily living (IADL), and functional mobility, respectively, while mental health was assessed as cognitive function and depression. The multi-criteria decision analysis (MCDA) (19) was applied to generate an aggregate index to represent the overall health status of the respondents, taking both their functional and mental health in the up-mentioned six aspects into account simultaneously. We hypothesized that social isolation would be associated with decreased physical and mental health and also the overall health status. Findings from the current study may provide new knowledge about the relationship of social isolation on physical function and mental health for the daily living among the elder adults who were current social care receivers. It informs future planning of targeted prevention and intervention initiatives to alleviate social isolation and promote healthy living in the older population.

## Materials and methods

### Study design, setting, and participants

**Figure 1:**
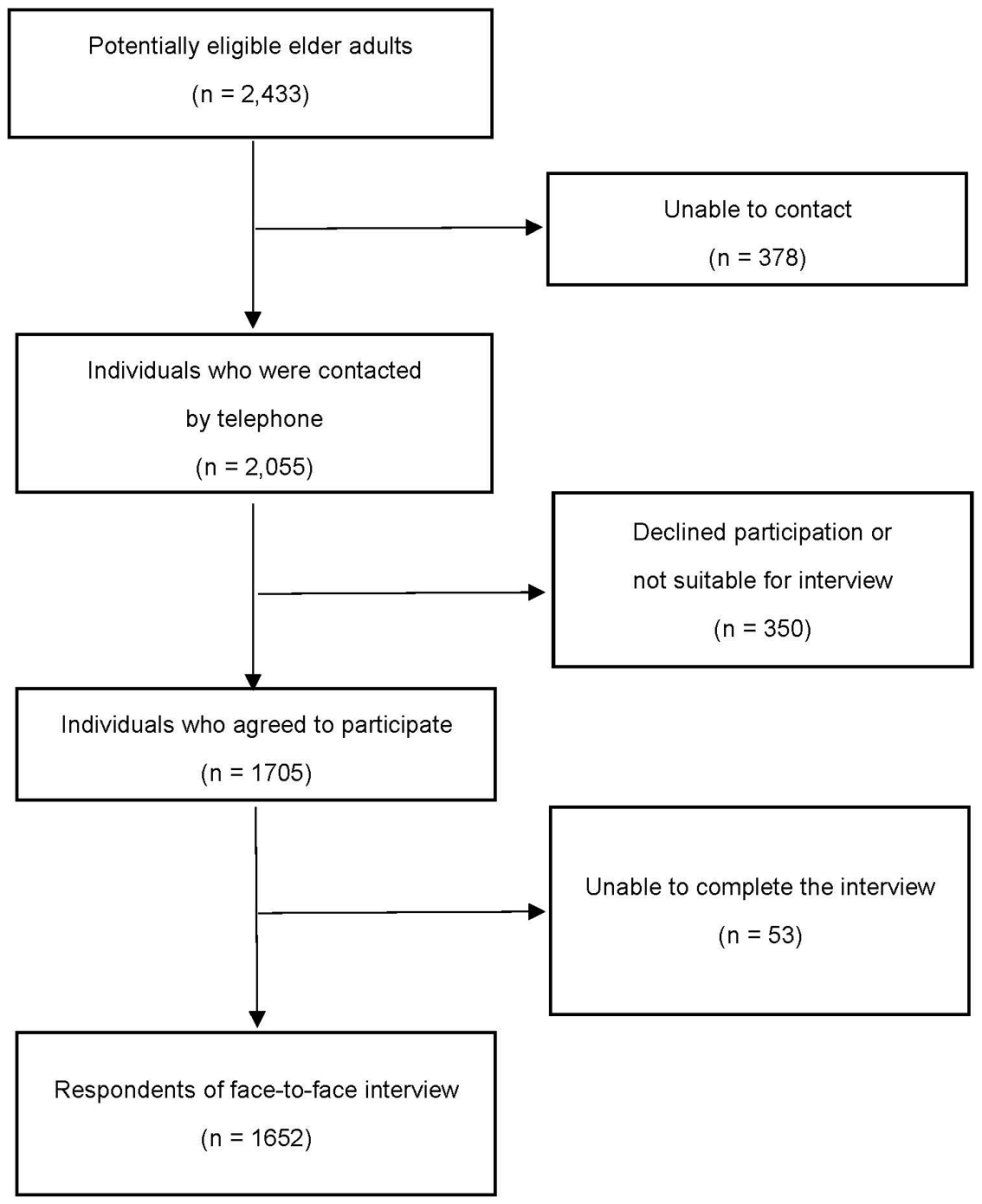
Flowchart of the recruitment process for participants in the study.

A cross-sectional face-to-face interview with a structured questionnaire was conducted in 2017–2018 among those older adults aged ≥60 and living in a community in the Kowloon Central District which is one of the high median ages in Hong Kong. The target elder adults were the members and also the social service recipients of the three NGOs serving in the Kowloon Central District area which was one of aging districts in Hong Kong. They were the Salvation Army in Yau Tsim Mong district, and Tung Wah Group of Hospitals in Homantin district, Ordinary Case of Hong Kong Christian Service in Shek Kip Mei district. The service recipients usually live alone or lack family support and they receive social support from the NGOs according to their needs, including meal delivery services, escort services, personal care, etc. Thus, convenience sampling was adopted with the assistance of the NGOs in the Kowloon Central District. Those diagnosed with dementia, Parkinson’s disease, or mentally incapacitated to response the survey were excluded from the survey. According to the initial sample size estimation, the minimum sample size is 1,050 if we hypothesized there would be 4 unit mean score difference of overall health status between the responses of the group with and without social isolation, with a standard deviation of 20 and at least 90% power to detect the statistical significance. A total of 2,433 potentially eligible elder adults were approached and 1,652 of them responded to the interview (Figure 1).

### Data collections

Active out-reach home-based service users of the Support Teams for the Older Adults and Integrated Home Care Services-Ordinary Cases were approached. The related organizational staff or volunteers got the initial verbal consent by telephone. The contact details of the potentially eligible individuals were passed to the research team for inviting the elder adults to participate in the study via telephone. Written consent was obtained before conducting the face-to-face interviews. Individuals who did not provide written consent were excluded from the study. Subjects who participated in the study were on a voluntary basis. Anonymity and confidentiality of the subject were maintained throughout the study. The project was approved by the Human Subjects Ethics Sub-Committee of the City University of Hong Kong.

### Measurement tools

The questionnaire consisted of three sections including social isolation, health status, and sociodemographics. The details of each section are provided in the following paragraphs.

*Social isolation* was assessed by the Lubben Social Network Scale-6 (LSNS-6), which is a validated instrument designed to gage social isolation in older adults by measuring the number and frequency of social contacts with friends and family members, and the perceived social support received from these sources (20). The scale contains two subscales: family [three items: “(1) How many relatives do you see or hear from at least once a month?”; “(2) How many relatives do you feel close to such that you could call on them for help?”; and “(3) How many relatives do you feel at ease with that you can talk about private matters?”], and friends (three items repeated by replacing the word *relatives* with the word *friends*). LSNS-6 score is an equally weighted sum of six items. Each item is rated on a six-point Likert scale. The total score ranges from 0 to 30, with the higher scores indicating a greater level of social support and a lower risk for isolation. A score of <12 means “at risk” for social isolation. LSNS-6 is a validated and reliable instrument and has been well applied among older adults of Chinese population (21).

*Health status* was assessed in six aspects: fall risk, cognitive function, depression, activities of daily living (ADL), instrumental activities of daily living (IADL), and functional mobility.

Fall risk was a binary variable measured by the Tinetti test. The test assesses the gait and balance of older adults. The lower the score on the Tinetti test, the higher the falling risk. In this study, a Tinetti score of >18 means “moderate/low fall risk”; ≤18 means “high fall risk” (22).

Cognitive function was a binary variable measured by the Mini-Cog test. It is a screening tool for the detection of cognitive impairment in older adults. A Mini-Cog score of ≥3 indicates a “lower likelihood of dementia” while ≤2 indicates a “higher likelihood of dementia” (23).

Depression was a binary variable measured by the shortened version of the Geriatric Depression Scale (GDS-15). It is a self-reported questionnaire to detect depression in older adults. A GDS-15 score of <8 is considered “normal” while ≥8 is suggestive of “depression” (24).

Performance in ADL was a numerical variable measured by the Barthel Index (BI), with a score ranging from 0 to 20. A higher score represents a greater ability to function independently (25). Performance in IADL including complex activities necessary for functioning in community settings (e.g., shopping, cooking, and managing finances) was a numerical variable measured by Lawton’s Instrumental Activities of Daily Living. The score ranges from 0 to 18, with a higher number indicating a greater ability to function independently (24). Functional mobility was a numerical variable measured by the Older Adults Mobility Score (EMS) ranging from 0 to 20, with a higher number reflecting more independent and safer functional mobility (26). A median score of BI, IADL, and EMS was used to categorize them into binary variables, with 0 indicating the normal (BI = 20, IADL≥16, and EMS = 20) and 1 the abnormal ADL, IADL, and dependency in functional mobility (BI<20, IADL<16, and EMS < 20), respectively.

Internal consistency reliability was examined for social isolation and health status, respectively, by Cronbach’s alpha coefficient. It is acceptable if Cronbach’s alpha is ≥0.7 (27).

*Sociodemographic* including age, gender, body mass index (BMI), education level [not educated, primary, secondary, and degree or above, marital status (married, widowed, divorced/separated, and single), current smoking and drinking behaviors (never and ever), living alone or not, with or without a caregiver, religion (yes and no), and type of housing (private and public, as a proxy of socioeconomics characteristics)] were collected and adjusted for possible confounding effects.

### Data analyses

Data description for the socio-demographic characteristics, social isolation, and health status: using frequency and proportion to present the categorical variables, and mean ± standard deviation (SD) to present the continuous variables. We used two independent samples T-test for continuous variables with approximately normal distribution, and a Chi-square test for categorical variables to compare the difference between two groups with or without social isolation.

We examined the associations of social isolation with health status, considering health status as the outcome variable and social isolation as the interpreted variable. As the distribution of BI, IADL, and EMS were all left-skewed (negatively skewed), we also transformed them into binary variables using the median as the cutoff point. Binary logistic regression with univariate and multivariate models was performed to examine the association, presenting the crude and adjusted odds ratio (OR) and corresponding 95% confidence intervals (CI). The sociodemographic characteristics including age, gender, BMI, marital status, ever smoking, ever drinking, living alone, religion, and housing type that may potentially confound the association were adjusted in the multivariate model.

An initial analysis showed that Cronbach’s alpha coefficient was 0.720 (0.705, 0.735) for six aspects of health indicators in the study. It implied that they were considered to have good internal consistency when put together as the measurement for health status. Thus, an aggregate index was estimated to represent the overall health score based on the self-reported physical function and mental health using multi-criteria decision analysis (MCDA) [19], taking six health indicators (fall risk, cognitive impairment, depression, BI, IADL, and EMS) into account simultaneously. Firstly, each of these six attributes (*x_ij_*) was normalized (*n_ij_*) using the following formula and determined the weight (*w_j_*) by the entropy-based method (28, 29). *i* and *j* denote the *i^th^* sample and the *j^th^* attribute.

The beneficial (the-bigger-the-better) attributes such as Tinetti, Mini-cog, BI, IADL, and EMS scores were normalized by:


$$n_{ij}=\frac{x_{ij}-\min\left(x_{ij}\right)}{\max\left(x_{ij}\right)-\min\left(x_{ij}\right)}$$

The non-beneficial (the-smaller-the-better) attribute such as the GDS score was normalized by:


$$n_{ij}=\frac{\max\left(x_{ij}\right)-x_{ij}}{\max\left(x_{ij}\right)-\min\left(x_{ij}\right)}$$

Then we calculated the entropy-based weight (*w_j_*) and applied the weighted sum method to aggregate the overall score of health status by:


$$Health\;statusscore_{i}=\overset{6}{\underset{j=1}{\sum }}w_{j}\times n_{ij}\times 100$$

The aggregate score of overall health status is a continuous variable ranging from 0 to 100, with the larger value denoting a better health status. As the overall health status score is a continuous outcome, we applied univariate and multivariate linear regression to examine the crude and adjusted associations of social isolation with the overall health status after adjusting the same sociodemographic characteristics as those in the up-mentioned multivariate logistic models. The regression coefficient represents the changes in overall health score that are associated with social isolation.

The significance test was two-sided and a value of *p* < 0.05 was considered to be statistically significant. Data processing and analyses were conducted in R version 4.1.2 (R Foundation for Statistical Computing, Vienna, Austria).

## Results

A total of 2,433 potentially eligible older adults were approached and 1,652 respondents conducted the interview. The recruitment flowchart is shown in Figure 1 for the details. However, only 1,616 participants answered the questions related to social isolation which were included in the study. Among them, 41.4% were identified as having social isolation. 66.3% were female with ages ranging from 61 to 104 and an average age of 80.9 years old. 40.1% of them were married, 47% were widowed, and 51.3% lived alone. The education level for 91.9% of the participants was secondary and below, with 21.3% ever smoking and 13.4% ever drinking. 59.1% of the participants had religions and 66.2% lived in public housing. Some sociodemographic characteristics were statistically significantly different between social isolation status, with higher proportions of males, divorced or unmarried, ever smoking and drinking, living alone, and living in public housing without religion in the socially isolated group compared to the non-isolated group (Table 1). The distribution of LSNS-6 scores was normal and the average score was higher in the non-isolated group than that in the socially isolated group (16.1 vs. 7.6).

**Table 1:** Sociodemographic characteristics for the participants between social isolation status (*N* = 1,616).

Variable	Total (*N* = 1,616)	Social Isolation		
		No (*N* = 947)	Yes (*N* = 669)	*p* value^*^
Age (years old), *mean ± SD*	80.9 ± 7.3	80.7 ± 6.9	81.0 ± 7.7	0.405
Gender, *n (%)*				**< 0.001**
Female	1,071 (66.3)	690 (72.9)	381 (57.0)	
Male	544 (33.7)	257 (27.1)	287 (43.0)	
BMI (Kg/m^2^), *mean ± SD*	23.3 ± 3.8	23.5 ± 3.8	23.0 ± 3.9	**0.016**
Marital status, *n (%)*				**< 0.001**
Married	645 (40.1)	401 (42.5)	244 (36.6)	
Widowed	756 (47.0)	462 (48.9)	294 (44.1)	
Divorced/Separated	108 (6.7)	45 (4.8)	63 (9.5)	
Unmarried	101 (6.3)	36 (3.8)	65 (9.8)	
Education level, *n (%)*				0.326
Not educated	358 (22.5)	214 (22.9)	144 (21.9)	
Primary	752 (47.2)	433 (46.4)	319 (48.4)	
Secondary	353 (22.2)	219 (23.4)	134 (20.3)	
Degree or above	98 (6.2)	50 (5.4)	48 (7.3)	
Other	32 (2.0)	18 (1.9)	14 (2.1)	
Ever smoking, *n (%)*				**< 0.001**
No	1,265 (78.3)	782 (82.6)	483 (72.2)	
Yes	344 (21.3)	164 (17.3)	180 (26.9)	
Ever drinking, *n (%)*				**0.003**
No	1,387 (85.8)	834 (88.1)	553 (82.7)	
Yes	217 (13.4)	107 (11.3)	110 (16.4)	
Living alone, *n (%)*				**0.011**
No	786 (48.7)	486 (51.4)	300 (44.8)	
Yes	828 (51.3)	459 (48.6)	369 (55.2)	
Had caregiver, *n (%)*				0.518
No	1,127 (70.2)	654 (69.5)	473 (71.1)	
Yes	479 (29.8)	287 (30.5)	192 (28.9)	
Was caregiver, *n (%)*				0.245
No	1,340 (84.3)	778 (83.4)	562 (85.7)	
Yes	249 (15.7)	155 (16.6)	94 (14.3)	
Religion, *n (%)*				**< 0.001**
No religion	642 (40.9)	334 (36.2)	308 (47.6)	
Had religion	927 (59.1)	588 (63.8)	339 (52.4)	
Housing type, *n (%)*				**< 0.001**
Private	547 (33.8)	365 (38.5)	182 (27.2)	
Public	1,069 (66.2)	582 (61.5)	487 (72.8)	
LSNS-6 score, *mean ± SD*	12.6 ± 5.3	16.1 ± 3.6	7.6 ± 2.8	**< 0.001**

* Two independent samples *t*-test is used for continuous variables and Chi-square test is used for categorical variables to compare the difference between two groups with or without social isolation.

Value of *p* < 0.05 is in bold.

The six health indicators including fall risk, cognitive impairment, depression, ADL, IADL, and functional mobility were all statistically significantly worse in the socially isolated group compared to the non-isolated group (Table 2). The proportions of high fall risk, cognitive impairment, and depression were significantly higher in the socially isolated group, while the proportions of high BI, IADL, and EMS scores were higher in the non-isolated group. The entropy-based method determined weights of 0.092, 0.541, 0.080, 0.034, 0.201, and 0.052 for the scores of Tinetti, Mini-cog, GDS, BI, IADL, and EMS, respectively. The weighted sum score denoted the overall health status score which was significantly higher in the non-isolated group than in the social isolation group (76.1 vs. 71.4; Table 2).

**Table 2:** Health status for the participants between social isolation status (*N* = 1,616).

Health status (Outcome variables)	Total (*N* = 1,616)	Social isolation		
		No (*N* = 947)	Yes (*N* = 669)	*p* value^*^
Fall risk (Tinetti)				
Moderate/low risk (Tinetti >18), *n (%)*	1,372 (84.9)	853 (90.1)	519 (77.6)	< 0.001
High risk (Tinetti ≤18), *n (%)*	194 (12.0)	76 (8.0)	118 (17.6)	
Cognitive impairment (Mini-cog)				0.008
Pass (Mini-cog ≥3), *n (%)*	1,107 (68.5)	683 (72.1)	424 (63.4)	
Fail (Mini-cog ≤2), *n (%)*	457 (28.3)	248 (26.2)	209 (31.2)	
Depression (GDS)				< 0.001
No depression (GDS < 8), *n (%)*	1,350 (83.5)	826 (87.2)	524 (78.3)	
Depression (GDS ≥ 8), *n (%)*	232 (14.4)	113 (11.9)	119 (17.8)	
Activities of daily living (BI)				
Normal (BI = 20), *n (%)*	1,211 (74.9)	775 (81.8)	436 (65.2)	< 0.001
Abnormal (BI <20), *n (%)*	403 (24.9)	172 (18.2)	231 (34.5)	
Instrumental activities of daily living (IADL)				
Normal (IADL ≥16), *n (%)*	1,047 (64.8)	670 (70.7)	377 (56.4)	< 0.001
Abnormal (IADL <16), *n (%)*	569 (35.2)	277 (29.3)	292 (43.6)	
Functional mobility (EMS)				
Normal (EMS = 20), *n (%)*	1,092 (67.6)	705 (74.4)	387 (57.8)	< 0.001
Dependency (EMS <20), *n (%)*	501 (31.0)	236 (24.9)	265 (39.6)	
Overall health status score, *mean ± SD*	74.2 ± 18.7	76.1 ± 17.5	71.4 ± 20.1	< 0.001

* Two independent samples *t*-test is used for continuous variable and Chi-square test is used for categorical variables to compare the difference between two groups with or without social isolation.

Table 3 shows the associations of social isolation with the six health indicators, using the univariate and backward stepwise logistic regression to estimate the crude and adjusted ORs with adjusting for the potential confounding from the socio-demographic characteristics. After adjusting for age, gender, BMI, marital status, ever smoking, ever drinking, living alone, religion, and housing type, the ORs comparing socially isolated vs. non-isolated groups were 2.52 (95%CI: 1.79, 3.56) for high fall risk, 1.51 (1.17, 1.94) for cognitive impairment, and 1.78 (1.31, 2.43) for depression. The socially isolated group increased the odds of abnormal BI, IADL, and EMS by 105–150% while comparing with the non-isolated group. When considering the six health indicators simultaneously, results from the multivariate linear regression showed that the socially isolated group may decrease the overall health status score by 5.30 (3.42, 7.18).

**Table 3:** Associations of Social isolation with Health status (*N* = 1,616).

Health status (Outcome variables)	Social isolation	
	Univariate model	Multivariate model^c^
Fall risk (Tinetti)^a^		
Moderate/low risk (Tinetti >18)	1.00	1.00
High risk (Tinetti ≤18)	2.55 (1.87, 3.47)	2.52 (1.79, 3.56)
Cognitive impairment (Mini-cog)^a^		
Pass (Mini-cog ≥3)	1.00	1.00
Fail (Mini-cog ≤2)	1.36 (1.09, 1.69)	1.51 (1.17, 1.94)
Depression (GDS)^a^		
No depression (GDS < 8)	1.00	1.00
Depression (GDS ≥ 8)	1.66 (1.25, 2.20)	1.78 (1.31, 2.43)
Activities of daily living (BI)^a^		
Normal (BI = 20)	1.00	1.00
Abnormal (BI <20)	2.39 (1.90, 3.00)	2.50 (1.92, 3.24)
Instrumental activities of daily living (IADL)^a^		
Normal (IADL ≥16)	1.00	1.00
Abnormal (IADL <16)	1.87 (1.52, 2.30)	2.05 (1.62, 2.60)
Functional mobility (EMS)^a^		
Normal (EMS = 20)	1.00	1.00
Dependency (EMS < 20)	2.05 (1.65, 2.54)	2.47 (1.93, 3.18)
Overall health status score^b^		
Continuous outcome variable	−4.65 (−6.57, −2.73)	−5.30 (−7.18, −3.42)

^a^Binary logistic regression and ^b^linear regression models are conducted to estimate the association of Social Isolation with Health Status, and the effect estimates are presented as ^a^OR with 95% CI and ^b^absolute change with 95% CI. ^c^The multivariate model is adjusted for sociodemographic characteristics including age, gender, BMI, marital status, ever smoking, ever drinking, living alone, religion, and housing type.

## Discussion

In the current cross-sectional survey among the old population who received social care services in Hong Kong, we estimated the prevalence of social isolation and its association with worse physical functions and mental disorders, which highlights the burden of social isolation on the health and well-being even among those old people with social care services.

The prevalence of social isolation identified in the study was 41.4%, which was severely higher than the 21.7% of social isolation identified in an old age group (70–79 years) of the German population before the COVID-19 pandemic (3), and comparative to a 41% classified social isolated in United Kingdom studies of participants aged 50 years and over (1, 2), but lower than the 61% of those aged 50 years or older who reported experiencing social isolation in a national survey conducted in the US in August 2020 since the COVID-19 pandemic began (4).

Our findings on the association of social isolation with both physical and mental health are consistent with the evidence in the literature. Fall risk in older people has been linked to social isolation or loneliness in a systematic review (8). A decrease in gait speed at follow-up and an increase in difficulties with activities of daily living were found to be associated with social isolation and loneliness in the English Longitudinal Studies of Aging (8, 9). And socially isolated participants were less likely to consistently report weekly moderate-to-vigorous physical activity over the 10 follow-up years than non-isolated participants among older adults (RR = 0.86; 0.77–0.97) (10). A China Health and Retirement Longitudinal Study also found that social isolation, rather than loneliness, was significantly associated with functional disability over 4 years among women—baseline social isolation was significantly associated with new-onset ADL [OR = 1.18 (1.07–1.30)] and IADL [OR = 1.11 (1.01–1.21)] disability (11). Evidence in the literature also supported the adverse effect of social isolation on mental disorders, including decreased cognitive function in later life (12–14), memory decline over time (15), depressive symptoms (2), and depression (16).

Our findings of social isolation as a major risk factor linked with poor physical and mental health status call for intervention to reduce social isolation in older people. Social isolation can be modified and the corresponding interventional program has been proposed in the literature. For example, video calls were supposed to help older people stay connected and reduce social isolation, and the effectiveness of video calls in reducing symptoms of depression and improving quality of life has been addressed (30). In a Spanish community setting, a multicomponent intervention comprising six domiciliary face-to-face sessions and five telephone calls was examined in a cluster randomized controlled clinical trial and its effectiveness in reducing social isolation and improving Health-Related Quality of Life has been proven (31). Approaches to address the issues of social isolation among older adults have also been proposed in the context of COVID-19, such as “promoting social connection as public health messaging, mobilizing the resources from family members, community-based networks and resources, developing innovative technology-based interventions to improve social connections, and engaging the health care system to begin the process of developing methods to identify social isolation in health care settings” (32). Furthermore, social isolation together with depressive symptoms may be frequently related to unsuccessful therapeutic responses. Evidence of buprenorphine at low doses as an efficacious, well-tolerated, and safe antidepressant pharmacotherapy has been documented (33).

The strength of the current study is that we add to the literature by applying multiple criteria decision analysis (MCDA) to create a comprehensive index to represent the overall health status, taking six attributes of physical functions and mental disorders into account simultaneously, and demonstrating the adverse impact of social isolation on overall health status. MCDA is a sub-discipline of operations research that explicitly evaluates multiple conflicting criteria in decision-making both in daily life and in settings such as business, government, and medicine. We demonstrated a good example by deciding on the overall health status from several attributes, which could be measured by the individual standardized instrument. This approach has also been applied to evaluate the performance of the healthcare system in low-and middle-income countries (29, 34).

There are a few limitations in this study. Due to the cross-sectional nature of this study, no causal relationships and underlying mechanisms can be examined. Mechanisms linking social isolation-related neurological dysfunctions with immune-inflammatory abnormalities have been documented in a systemic review (35). Future studies can utilize a longitudinal design to examine how social isolation may influence health status or vice versa over time. Second, we did not collect information on the type of home care services while the type of services received (e.g., meal delivery vs. escort) may differ in the frequency and duration of social interaction with volunteers; therefore, the potential differences in social isolation by the type of home care services could not be assessed. Finally, the prevalence of social isolation identified in this study is from a convenience sample of old participants who received social care services from all three NGOs serving for the Kowloon central districts of Hong Kong. The social service providers may potentially help to address the high prevalence of social isolation observed in this population, and the prevalence may vary in different districts due to the services type and different profiles of demographic characteristics, thus limiting the generalization of the findings on social isolation prevalence in an overall Hong Kong elder adult population. However, it indicates an alert to the need for a new social service model for targeted prevention and intervention initiatives among community-dwelling old adults to alleviate social isolation for better health and good functioning in the community. In addition, the association of social isolation with the poorer overall health status demonstrated in this study may suggest the overall negative health impact of social isolation in the old population.

In conclusion, we demonstrated the association of social isolation with poorer functional and mental health and overall health status. These findings highlight the need for targeted prevention and intervention initiatives to alleviate social isolation. Future studies may shed the light on the casual effect between social isolation and health status in different populations and also the new design of social services for improving the problem.

## Data availability statement

The datasets generated and/or analysed in the current study are available upon request.

## Ethics statement

The studies involving human participants were reviewed and approved by the Human Subjects Ethics Sub-Committee at the City University of Hong Kong. The patients/participants provided their written informed consent to participate in this study.

## Author contributions

FC obtained the research funding. The study design was developed by FC, EW, and E-KY. HL supervised the data collection and managed the data, including quality control. HQ provided statistical analyses of the data. The preparation of the manuscript was performed by EW, HQ, and AC, and participated by FC. EW takes responsibility for the paper as a whole. All authors contributed to the article and approved the submitted version.

## Funding

This study received support from Dr. Joseph Lee Chung-tak of Wofoo Social Enterprises and Mr. Lau Tai-chuen of Sino International Industrial Limited. The funders were not involved in the study design, collection, analysis, interpretation of data, the writing of this article, or the decision to submit it for publication. The authors are grateful for their generous financial support.

## Conflict of interest

The authors declare that the research was conducted in the absence of any commercial or financial relationships that could be construed as a potential conflict of interest.

## Publisher’s note

All claims expressed in this article are solely those of the authors and do not necessarily represent those of their affiliated organizations, or those of the publisher, the editors and the reviewers. Any product that may be evaluated in this article, or claim that may be made by its manufacturer, is not guaranteed or endorsed by the publisher.

## Abbreviations

ADL: Activities of daily life
BI: Barthel index
CI: Confidence interval
EMS: Elderly mobility score
GDS: Geriatric depression scale
IADL: Instrumental activities of daily living
LSNS-6: Lubben social network scale-6
MCDA: Multi-criteria decision analysis
OR: Odds ratio
